# Biocalcite, a multifunctional inorganic polymer: Building block for calcareous sponge spicules and bioseed for the synthesis of calcium phosphate-based bone

**DOI:** 10.3762/bjnano.5.72

**Published:** 2014-05-12

**Authors:** Xiaohong Wang, Heinz C Schröder, Werner E G Müller

**Affiliations:** 1 ERC Advanced Investigator Grant Research Group at Institute for Physiological Chemistry, University Medical Center of the Johannes Gutenberg University Mainz, Duesbergweg 6, D-55128 Mainz, Germany

**Keywords:** biocalcite, bioprinting, bone, bone formation, calcareous spicules, sponge

## Abstract

Calcium carbonate is the material that builds up the spicules of the calcareous sponges. Recent results revealed that the calcium carbonate/biocalcite-based spicular skeleton of these animals is formed through an enzymatic mechanism, such as the skeleton of the siliceous sponges, evolutionarily the oldest animals that consist of biosilica. The enzyme that mediates the calcium carbonate deposition has been identified as a carbonic anhydrase (CA) and has been cloned from the calcareous sponge species *Sycon raphanus*. Calcium carbonate deposits are also found in vertebrate bones besides the main constituent, calcium phosphate/hydroxyapatite (HA). Evidence has been presented that during the initial phase of HA synthesis poorly crystalline carbonated apatite is deposited. Recent data summarized here indicate that during early bone formation calcium carbonate deposits enzymatically formed by CA, act as potential bioseeds for the precipitation of calcium phosphate mineral onto bone-forming osteoblasts. Two different calcium carbonate phases have been found during CA-driven enzymatic calcium carbonate deposition in in vitro assays: calcite crystals and round-shaped vaterite deposits*.* The CA provides a new target of potential anabolic agents for treatment of bone diseases; a first CA activator stimulating the CA-driven calcium carbonate deposition has been identified. In addition, the CA-driven calcium carbonate crystal formation can be frozen at the vaterite state in the presence of silintaphin-2, an aspartic acid/glutamic acid-rich sponge-specific protein. The discovery that calcium carbonate crystals act as bioseeds in human bone formation may allow the development of novel biomimetic scaffolds for bone tissue engineering. Na-alginate hydrogels, enriched with biosilica, have recently been demonstrated as a suitable matrix to embed bone forming cells for rapid prototyping bioprinting/3D cell printing applications.

## Introduction

The size and complexity of a metazoan taxon is correlated with the dimensioning of its respective complex composite skeleton. This statement can be exemplarily illustrated by comparing different sponge [phylum: Porifera] species. These animals are grouped to the classes of the siliceous sponges, the Hexactinellida and the Demospongia, and the calcareous sponges, the Calcarea [1–2]. Sponges of the ‘‘crusty’’ asconoid type Calcarea, e.g., the encrusting *Clathrina coriacea*, measure about 2 mm in thickness, the syconoid type calcareous sponges, e.g., *Sycon raphanus* have an axis length of 1–5 cm, while species with a leuconoid type Bauplan can be up to 3 m in size, e.g., the demosponge *Spheciospongia vesparium*, or the hexactinellid *Monorhaphis chuni* [1–2]. The evolutionary oldest animals on earth to comprise a skeleton formed of biosilica and are found among the siliceous sponges, the Hexactinellida and later in the Demospongiae [3–4], while the mineralized skeletons of the calcareous sponges are built of calcite [5]. The selection of the mineral appears to parallel the levels of silicate and carbonate in the marine environment [6].

The distinguished feature of biosilica-based skeletons is the fact that this polymer is formed enzymatically, a finding that resulted in the introduction of a new paradigm in biochemistry that also *inorganic polymers* and not only *organic polymers* can be formed enzymatically from their respective precursors [7]. This first clue, together with the finding that all animals, including the sponges, are of monophyletic origin gave the basis for the view that the bodyplan of the metazoans follows more universal genetic blueprints and, in turn, more general biochemical relationships [8].

Following this intellectual approach we asked the question, does the evolutionary oldest inorganic polymer, biosilica, share a functional relationship with the skeletal elements of the crown mammals, the calcium phosphate/hydroxyapatite (HA)-based skeletal systems. The understanding of the genetic blueprint of any morphogenetic event must begin with the identification and functional characterization of the individual expressed genes (proteins), followed by the elucidation of the interaction of the proteins, e.g., acting during mineralization, in other words, should start with the disclosure of the regulatory network of the proteins involved. It should be the aim to unravel the regulatory genetically-controlled architecture of proteins, based on the expression of a few molecules, and to pinpoint a single master gene that, after switching on, initiates the direction of development of a structural skeletal element.

**Figure 1:**
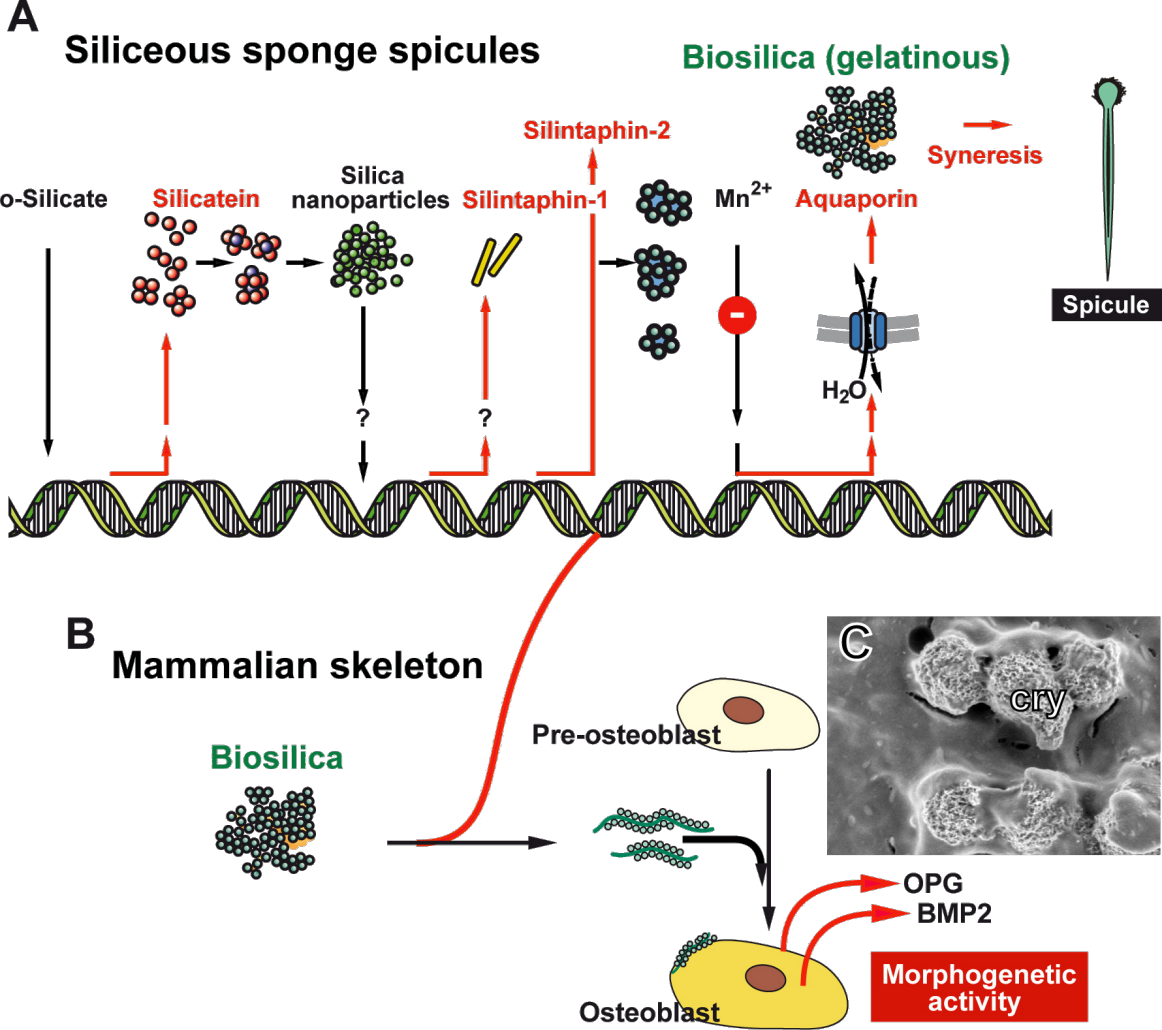
Function of biosilica during (A) the formation of siliceous sponge spicules and (B) mammalian bone mineral formation; scheme. (A) The different key molecules involved in spicule formation are sequentially expressed [11]. o-Silicate induces the expression of silicatein, followed by the expression of silintaphin-1 and silintaphin-2, a process that is most likely induced by biosilica nanoparticles. These two classes of molecules (silicateins and silintaphins) form mesoscopic gelatinous flocs that harden through syneresis to spicules [15]. The latter process is driven by water extrusion via aquaporin channels. (B) Effect of biosilica on mammalian bone-forming cells (SaOS-2 cells) in vitro. After incubation with biosilica those cells differentiate and express osteoprotegerin (OPG) and the bone morphogenetic protein 2 (BMP2), two components of their newly acquired morphogenetic activities. (C) SaOS-2 cells that have formed calcium-based carbonate and phosphate crystallites (cry).

Only recently it was possible to describe the molecular level of the formation of a hard skeleton (reviewed in [4]). Initial investigations were successfully performed with the siliceous sponge spicules. The key discovery was the identification of silicatein, the enzyme that initiates the biocatalytic biosilica-condensation reaction [9–11]. It initiated the resolution of the biochemical processes leading to biosilica formation (Figure 1A). The silicateins are members of the cathepsin L and papain family of proteases. They have been discovered in the demosponge *Tethya aurantium* by the group of Morse [9–10] and soon thereafter were also identified in the demosponge *Suberites domuncula* [11]. Based on biochemical studies, three isoforms of silicatein have been described in *T. aurantium*, silicatein-α to -γ. They have similar molecular weights (approximately 34 kDa). Among them the silicatein-α is the dominant isoform, forming the axial filament, residing in the axial canal. In *T. aurantium* the molar ratio between silicatein-α and silicatein-β was determined to be 2:1, while in *S. domuncula* the molar ratio amounts to 4:1. Soon after the expression of the silicateins and after the first formation of silica nanoparticles, the silicatein-interacting proteins, the silintaphins, are read out (Figure 1A). Until now two silintaphins, silintaphin-1 [12] and silintaphin-2 [4], have been described extensively. Silintaphin-1 significantly enhances the biosilica-forming activity of silicatein in vitro. A 5.3-fold increase of the biosilica-forming activity is measured at a molar ratio of 4:1 [silicatein-α/silintaphin-1] [13]. Likewise, in *S. domuncula* the 15-kDa protein silintaphin-2 had been identified as a second silicatein-interactor. Like silintaphin-1, this protein is located in the axial filament, but particularly in the organic cylinder around the growing spicules. Silintaphin-2 is a Ca^2+^-binding protein that complexes four Ca^2+^ ions [14].

The formation of HA and HA resorption by osteoclasts and osteoblasts in bone tissue are controlled by a network of cytokines and growth factors. The receptor activator of NF-KB (RANK) and its ligand, receptor activator of NF-KB ligand (RANKL) and osteoprotegerin (OPG) play a key function in regulation of bone formation and bone degradation (reviewed in [16]). The molecular triad OPG/RANKL/RANK not only regulates the differentiation of osteoclasts, but also differentiation processes in the vascular system and in the immune system. Addition of biosilica leads to an increased expression of OPG. The consequence is an inhibition of the differentiation of osteoclasts from their respective precursor cells (Figure 1B). In addition, after exposure of SaOS-2 cells to biosilica these cells increase the synthesis of the bone morphogenetic protein 2 (BMP2), a cytokine that induces osteoblast differentiation and mineralization (Figure 1C) (see [17–18] and reviewed in [16]).

## Review

### Calcium carbonate/bicarbonate a potential bioseed for Ca phosphate mineral formation by osteoblasts

It is well established that the calcium salt of carbonate is an effective diet supplement for amelioration of bone-loss during postmenopausal osteoporosis [19]. Recently, we could show in in vitro studies, by using SaOS-2 cells growing in calcium bicarbonate-deprived medium that these cells respond with a significant increase in calcium deposit formation after exposure to bicarbonate [20]. The cells start to form larger crystallite nodules on their surfaces, compared to the controls. Of course, the prerequisite has to be fulfilled that simultaneously with bicarbonate the cells have to be treated with the mineralization activation cocktail (MAC), composed of β-glycerophosphate, ascorbic acid and dexamethasone. One plausible explanation that emerged from this observation was that those crystalloids, which have been formed metabolically, are composed of calcium carbonate. This assumption was corroborated by the well established finding that mammalian skeletons contain, besides of HA-composed bones, biomineralized otoliths in the vestibular labyrinth of the vertebrate ear. There the inorganic matrix consists to 90 to 95% of calcium carbonate in the aragonite form [21–22]. Not only in those skeletal elements but also in the vertebrate bones calcium carbonate co-exists to a considerable amount with Ca phosphate [23].

Under physiological conditions the major processes of biomineralization of bone, teeth and otoconia proceeding in vertebrates mainly occur extracellularly, while intracellular mineral deposits are predominantly assembled during pathological calcifications of soft tissues [24]. Bone formation is based on a tightly controlled process between osteoblasts and fibrillar organic structures that starts from collagen fibrils around which poorly crystalline carbonated apatite aggregates are deposited [25–26]. Carbonated apatite are deposits in which carbonate ions (CO_3_^2−^) reversibly substitute either phosphate (PO_4_^3−^) or hydroxyl (OH^−^). Analyses by X-ray and electron diffraction and Fourier transform infrared spectroscopy, as well as determination of the chemical composition revealed that at least under in vitro conditions in osteoblasts low concentrations of carbonate ions exist in their Ca^2+^/phosphate mineral phase. Parallel spectroscopic studies suggested that Ca-deposition in osteoblasts starts intracellularly in calcium-rich vesicles that substantially contribute to the formation of bone apatite [27].

Both calcium phosphate formation [28] and calcium carbonate deposition [29] are exergonic processes that, in turn, are thermodynamically possible, but are strictly controlled in a biochemical system through the activation energy barriers that prevent a chemical reaction from occurring at physiological temperatures/conditions [30]. A modulation of the activation energy barriers enables an organism to control under which physiological conditions a thermodynamically possible reaction can be initiated or prevented [30]. Almost exclusively, alterations of the heights of the activation energy barriers are adjusted by enzymes or by the surface architecture of membranes separating two phases. The recent findings that in animals the inorganic polymer biosilica (see: [4,7]) is synthesized enzymatically through silicatein prompted us to study if also during calcium carbonate deposition there is an enzyme-driven step involved [31].

### Carbonic anhydrase, the basis for the accelerated calcium carbonate synthesis in calcareous sponges

The prevalent enzymes that allow bicarbonate to be formed in an organism are the carbonic anhydrases [CAs] [32]. These enzymes, which are characterized by an extremely high turnover number, catalyze the reversible hydration of carbon dioxide (CO_2_) to bicarbonate. This reaction occurs also in the presence of Ca ions [33]. Four of the seven metazoan CA isoenzymes are cytosolic, CA-I, -II, -III, and -VII. Among them the CA-II is a widely studied one [34]. Recently, experimental evidences have been presented revealing that the CAs might be involved in bone formation [35]. The mammalian CA-II, a cytosolic enzyme, is targeted in intact cell systems under certain physiological conditions to the cell membrane [36–37].

**Figure 2:**
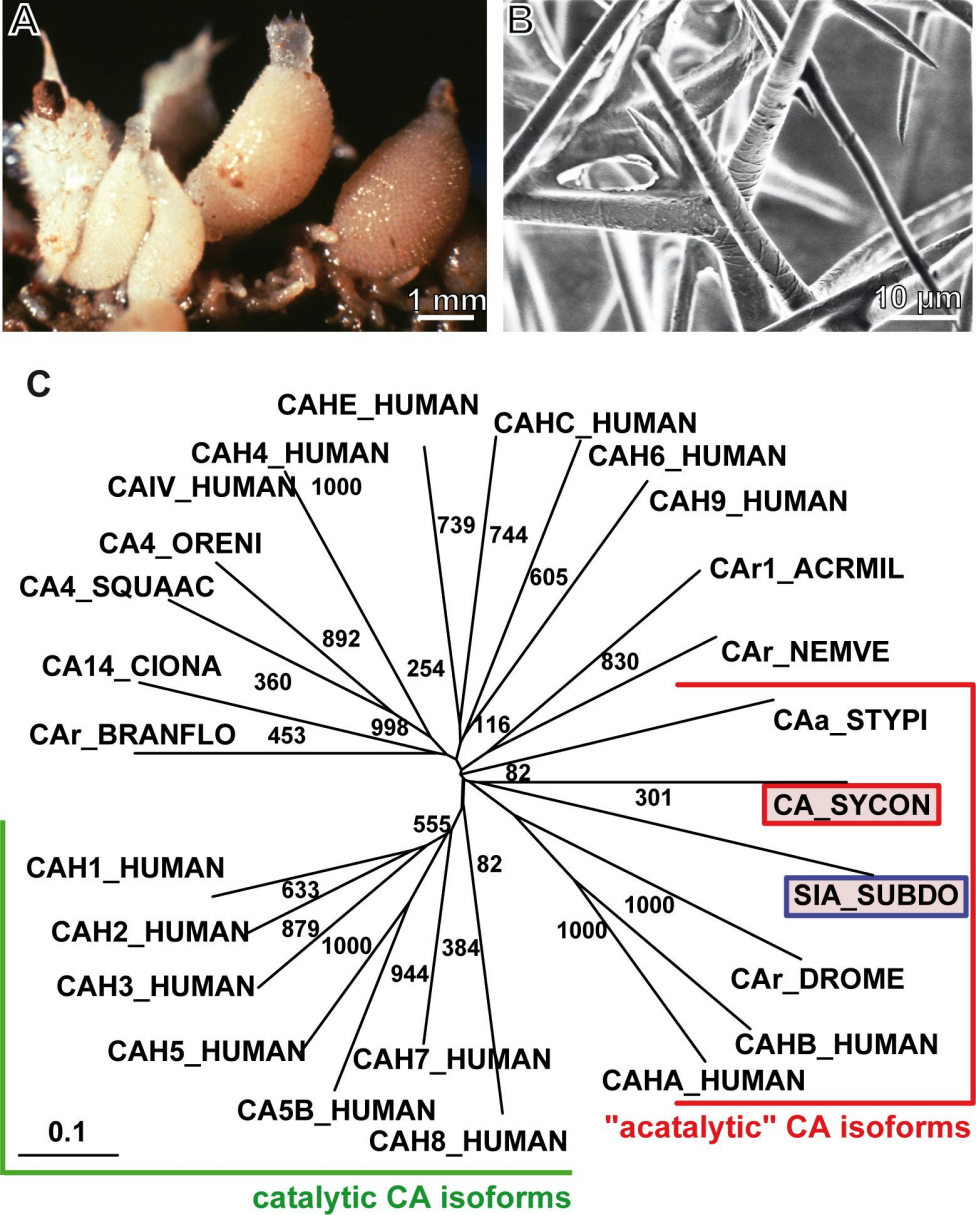
*Sycon raphanus*, its spicules and its CA. (A) Specimens of *S. raphanus*; (B) the calcareous spicules. (C) Phylogenetic, radial tree computed with the putative calcareous sponge *S. raphanus* carbonic anhydrase (CA_SYCON; accession number CCE46072) and the demosponge *S. domuncula* silicase (SIA_SUBDO; DD298191), as well as the carbonic anhydrase isoforms from human: I (CA-I) (CAH1_HUMAN; P00915); II (CA-II) (CAH2_HUMAN; P00918); II-2 (CA II) (CAHB_HUMAN; O75493), III (CA-III) (CAH3_HUMAN; P07451); IV (CAIV_HUMAN; AAA35625.1); IV (CA-IV) (CAH4_HUMAN; P22748); VA (CAH5_HUMAN; P35218); VB (CA5B_HUMAN; O75493); VI (CA-VI) (CAH6_HUMAN; P23280); VII (CA-VII) (CAH7_HUMAN; P43166); VIII (CA-VIII) (CAH8_HUMAN; P35219); IX (CA-IX) (CAH9_HUMAN; Q16790); X (CA-RP X) (CAHA_HUMAN; Q9NS85); XII (CA-XII) (CAHC_HUMAN; O43570); XIV (CA-XIV) (CAHE_HUMAN; Q9ULX7). In addition, the related sequences from the scleractinian *Acropora millepora*-1 (CAr1_ACRMIL; ACJ64662.1), the stony coral *Stylophora pistillata* (CAa_STYPI; ACA53457.1, EU159467.1), the anthozoan *Nematostella vectensis* (CAr_NEMVE; XP_001627923.1), the tunicate *Ciona intestinalis* (CA14_CIONA; XP_002123314.1), the lancelet *Branchiostoma floridae* (CAr_BRANFLO; XP_002601262.1), the shark *Squalus acanthias* (CA4_SQUAAC; AAZ03744.1), the fish *Oreochromis niloticus* (CA4_ORENI; XP_003456174.1), and the insect enzyme from *Drosophila melanogaster* (CAr_DROME; NP_572407.3) are included. The CAs, belonging to the "acatalytic" CA isoforms and of the catalytic CA isoforms, are surrounded. Partially taken from [38] with permission.

Among the phylogenetically oldest animals that have a skeleton based on calcium carbonate are the calcareous sponges with *Sycon raphanus* as an example (Figure 2A), the CA enzyme was cloned, expressed and functionally tested [38]. From this organism the complete cDNA encoding for the CA was obtained (accession number CCE46072). The complete 1,476 nts cDNA encodes, within its ORF [open reading frame] (from nt_68–70_ to nt_1001–1003_), the 312 aa putative CA, having a *M*_r_ of 33,251 and an pI of 5.81. The closest human related CA to the *Sycon* enzyme (Figure 2C), the human CA-X (CAHA_HUMAN; Q9NS85), and -XI (CAHB_HUMAN; O75493), are grouped to the "acatalytic" CA isoforms of unknown function, which have been proposed to be devoid of CO_2_ hydration activity [39]. However, the branch with the two sponge CAs comprises a common origin with the stony coral *Stylophora pistillata* enzyme [40] for which an enzymatic activity has been proven. Those human CAes, comprising CO_2_ hydration activity (the catalytic CA), are in the CA group I to IV.

**Figure 3:**
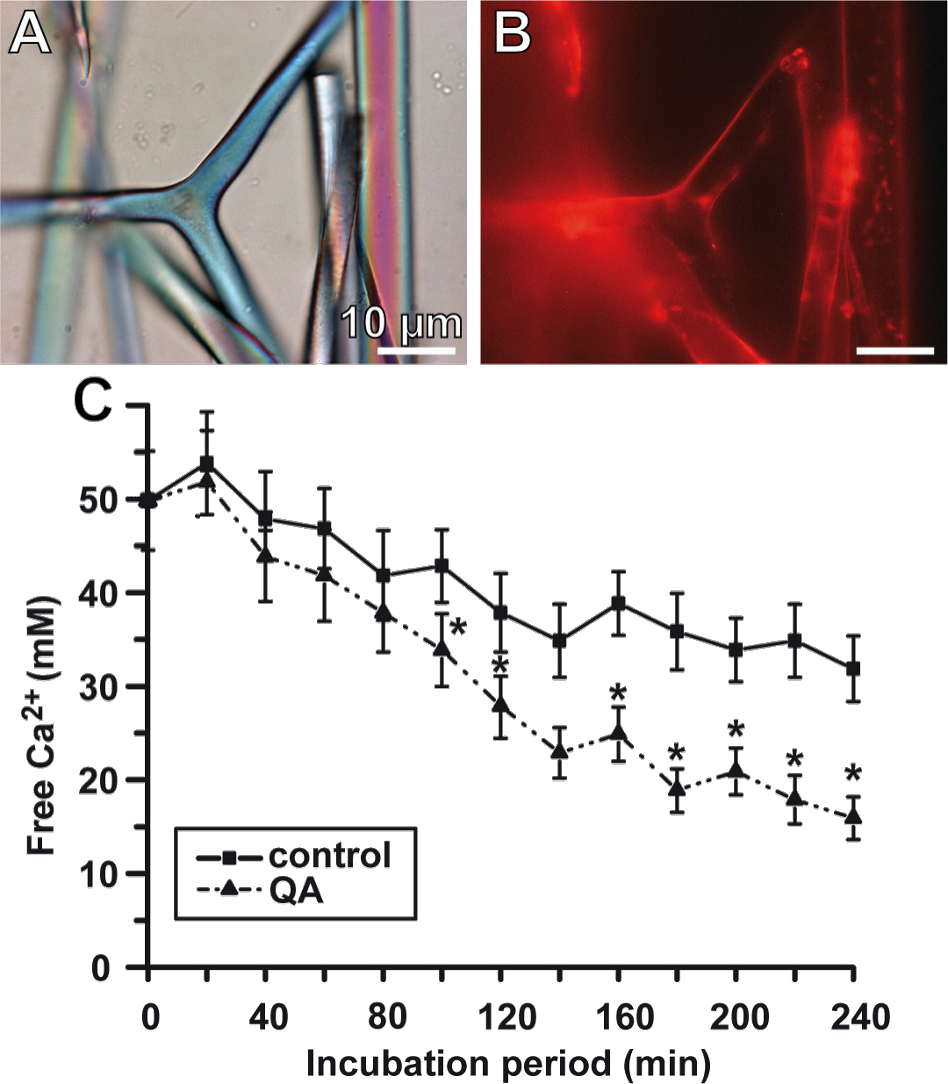
*Sycon* CA, its localization and in vitro function. Reacting of *Sycon* spicule with antibodies, raised against the homologous CA. (A) Light microscopic image of the spicules (in the center is a large triactine). (B) The spicules have been reacted with polyclonal antibodies, raised against the *Sycon* CA. The immunocomplexes were stained with Cy5-labelled anti-rabbit IgG. (C) Formation of CaCO_3_ in the ammonium carbonate diffusion assay in the presence of CA. For this series of experiments the recombinant human CA2 enzyme, expressed in *Escherichia coli* (C6624, Sigma), with a specific activity of about 5,000 units/mg, was added at a concentration of 35 W-A units (10 μg)/500 μL of CaCl_2_ to the assays. The formation of calcium carbonate was determined quantitatively on the basis of the consumption of free Ca^2+^ ions using the EDTA titration procedure [31]. The assays either remained free of additional compound(s) (filled square) or were supplemented with 10 μM quinolinic acid (QA, filled triangle). Samples of six parallel determinations were quantitated; means ± SD are given. *p < 0.05.

The Zn-binding sites that are involved in the catalytic reaction (hydration of CO_2_) are present in the CA-alpha (vertebrate-like) group stretch of the *S. raphanus* protein. The Zn ions are bound to the enzyme through the three His residues in the catalytic center of the enzyme [41]. The presence of a signal peptide cleavage site in the sponge CA indicates that this enzyme is secreted by the sponge cells or bound to the cell membrane. The spicules from the calcareous sponges (Figure 2B), e.g., *Sycon* used in our studies [38,42], consists of almost pure calcium carbonate (calcite). In a first approach to investigate the formation of the calcareous spicules on the molecular level, the function of the CA in this process has been studied by using *S. raphanus* as a model. The cDNA of the *Sycon* CA was prepared in a recombinant way and used to raise antibodies. Immunostructural studies revealed that the *Sycon* CA is localized on the surface of mature, developed spicules, the ca. 300 µm long diactines and the ca. 300 µm large triactines and tetractines (Figure 3A and B). It is assumed that the membranous, organic sheaths described to cover the spicules [43–44] are composed predominantly of this enzyme. Subsequently the recombinant enzyme was used to determine the in vitro calcium carbonate formation by applying the in vitro diffusion assay [45].

Even though the present-day oceans are supersaturated with respect to calcium carbonate, only very rarely spontaneous abiotic precipitation is seen [46]. Like in other metazoan systems, e.g., mollusks or echinoderms, in sponges carbonate is taken up from the aqueous environment as bicarbonate via specific membrane transporters, characterized by a Michaelis–Menten constant of around 50 mM [47], or is produced metabolically. At this concentration, calcium carbonate precipitates at an extent of about 50% during an incubation period of only 20 h in an ammonium carbonate diffusion/“dessicator assay” at pH 7–8 [48]. However, this reaction velocity is too slow to account for the rate of calcium carbonate deposition measured in vivo, e.g., in the sponge spicule formation in *Sycon* sp. [49]. The *Sycon* spicules, with a diameter of about 4 μm and a length greater than 100 µm, show a very fast growth rate of 65 μm/h. Hence it has to be concluded that an acceleration of the velocity of the exergonic reaction at ambient environmental conditions has to occur by lowering the activation energy by an enzyme, or by allowing the calcium carbonate process to proceed on a functionalized organic surface. In our previous studies we tested the first possibility [7,31].

As the substrate for the enzymatic reactions in the “dessicator assay” we used a solution of 50 mM CaCl_2_ over which CO_2_ vapor, generated from NH_4_HCO_3_ solution, was passed. The pH of the reactions was adjusted to 7.5 [7,31]. The mineralization process (based on the decrease of free Ca^2+^ concentration measured) started after an initial lag phase of 5 h. Addition of the recombinant CA (35 W-A units/500 μL) significantly increased the reaction velocity and accelerated the mineralization process; after 50 min already 25% of the CaCl_2_ had been precipitated, in the presence of CO_2_, to calcium carbonate (Figure 3C). An extent of 80% of precipitated calcium carbonate was reached after 10 h. To highlight again, the major role of the CA during the stages of enzymatic synthesis of calcium carbonate is to accelerate the reaction velocity, an essential feature of any enzyme. Furthermore, the CA allows the process of calcium carbonate deposition to occur at ambient physiological conditions.

**Figure 4:**
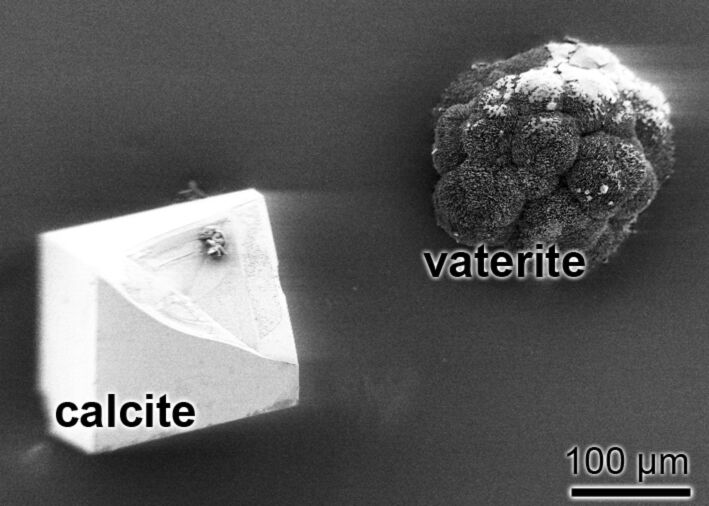
Calcium carbonate crystals formed in vitro (ammonium carbonate diffusion assay) by using *Sycon* CA. Left: a calcite crystal formed; at the right a vaterite crystal that has been formed.

Two morphologically different deposits are formed in the in vitro assay in the presence of the CA (35 W-A CA units per assay): first prisms with an average size of 80–120 μm and second round-shaped deposits of similar dimensions (Figure 4). Predominantly prisms with a rhombohedral morphology are formed, which are composed of calcite, as analyzed by MIDAC Fourier transform infrared spectroscopy (the characteristic vibrational bands at 873 cm^−1^ and 711 cm^−1^; Figure 4, left crystal). Intermediately, during the formation of the calcite crystals, round-shaped vaterite deposits are formed (875 cm^−1^ and 744 cm^−1^; Figure 4, right crystal). It is remarkable and likewise indicative that the biogenic CA-driven calcium carbonate crystal formation can be frozen at the vaterite state (in spite of the overall thermodynamically possible “end-point” transition formation to calcite) if the CA-driven reaction proceeds in the presence of silintaphin-2, a sponge-specific protein that is rich in aspartic acid (Asp, D) and glutamic acid (Glu, E) [50]. The hardness, elastic modulus and creep of the two forms of the calcium carbonate deposits, the calcitic prisms and the round-shaped vaterite deposits were determined by nanoindentation. The load–displacement curves obtained for the two calcium carbonate forms revealed the following values: for the rhombohedral calcite 1.98 ± 0.31 GPa and for the round-shaped vaterite deposits only 1.38 ± 0.39 GPa. Concurrently, a distinct decrease of the elastic modulus was measured for the vaterite deposits (39.13 ± 8.04 GPa), in comparison to the rhombohedral calcite prisms (72.83 ± 11.68 GPa). This significant difference in the mechanical properties between the two morphologies can also be deduced from the creep behavior. While the creep characteristics for the rhombohedral calcitic prisms was found to be 5.44 ± 1.15 (per maximal depth [%]), the corresponding value for the round-shaped vaterite deposits is 9.95 ± 1.60.

The enzyme-mediated deposition of calcium carbonate is markedly temperature dependent [31]. While at 10 °C the reaction velocity of calcium carbonate deposition is almost identical in the enzyme-containing and enzyme-lacking assays, at higher, physiologically more relevant incubation temperatures (e.g., 22 °C [42]), the reaction velocity of the CA-driven calcium carbonate formation is significantly higher (about 2-fold) than that in the absence of CA. Varying the pH value in the precipitation assay shows that in the absence of CA the precipitation of calcium carbonate increases only slightly from pH 6.0 to pH 8.1. In contrast, the CA-driven reaction velocity increases markedly (by over 5-fold) from pH 6.0 to pH 8.0. Importantly, the increased rate in the reaction velocities seen in the CA-containing assays can be inhibited almost completely by the CA-specific inhibitor acetazolamide at 3 μM. In those assays the calcium concentration had been 50 mM with respect to CaCl_2_ [31]. These findings are compatible with the view that the calcium carbonate deposition in the system described is enzymatically driven by the CA.

In order to underscore the dominant enzymatic contribution to the calcium carbonate deposition in vitro, one kinetic characteristic, the Michaelis–Menten constant, for CA, was determined [31]. At first it should be mentioned that the reaction follows substrate saturation kinetics. Under the assay conditions used (50 mM CaCl_2_, pH 7.5, 25 °C), the linear increase of the reaction velocity is seen between 0 and 20 mM CaCl_2_. Only at higher concentrations a saturation level is approached. It is well established that the CAs function both as hydratase, in the formation of bicarbonate, and also as esterase [51]. The Michaelis–Menten constants (*K*_m_) for both reactions are almost identical at around 5 mM for the hydratase (using CO_2_ as substrate) and for the esterase (with the substrate 4-nitrophenylacetate). The constant *K*_m_ for the sponge CA/esterase was determined by applying the method of Lineweaver and Burk. The apparent *K*_m_ constant for the sponge recombinant CA using 4-nitrophenylacetate as esterase substrate was found to be 6.2 ± 1.0 mM, at a maximal reaction velocity of 0.32 ± 0.05 mmol·mL^−1^·min^−1^. Using the same approach, the apparent *K*_m_ constant in the hydratase/CO_2_ diffusion assay was calculated to be 9.9 ± 2.1 mM (with respect to CaCl_2_) at a corresponding *v*_max_ of 24.9 ± 3.7 mmol·mL^−1^·min^−1^.

### Carbonic anhydrase: Evidence for forming bioseeds during mammalian hydroxyapatite formation

**Figure 5:**
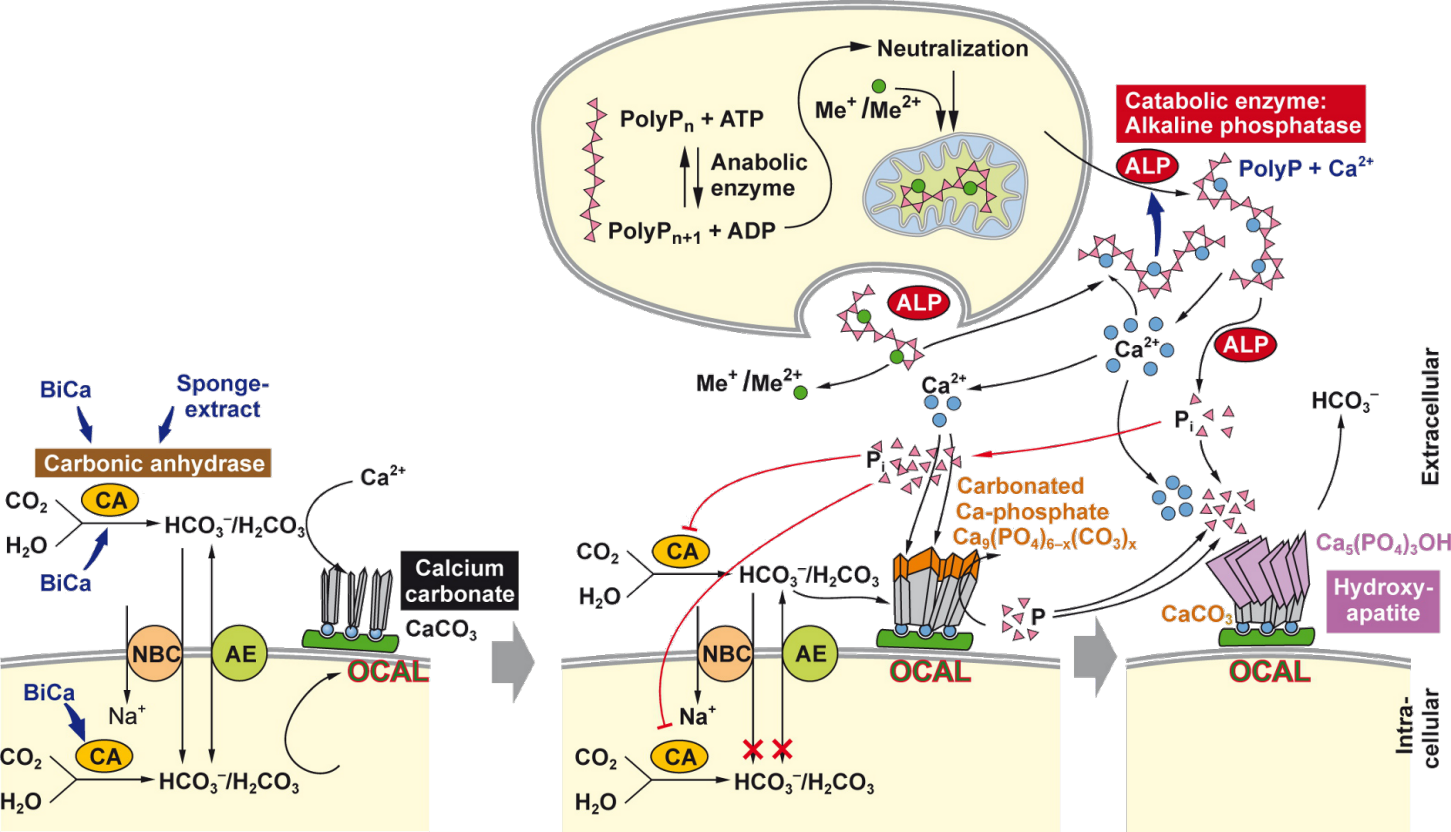
Sketch proposing the sequential deposition of calcium carbonate and Ca phosphate on the surface of bone-forming cells (SaOS-2 cells). The CA drives/accelerates the formation of bicarbonate, which reacts to carbonic acid and finally undergoes precipitation to calcium carbonate. Bicarbonate is provided to the CA via the chloride/bicarbonate anion exchanger (AE), or by the sodium bicarbonate co-transporter (NBC). It is proposed that the calcium carbonate crystallites are formed in the vicinity of the plasma membrane, perhaps under the participation of osteocalcin (OCAL). Sponge extracts (as well as their components, e.g., quinolinic acid) and bicarbonate (abbreviated as BiCa) stimulate the CA. In the second step calcium phosphate precipitates in the extracellular space onto the calcium carbonate bioseeds under formation of calcium carbonated apatite. Finally, orthophosphate, released from polyphosphate (polyP), downregulates the activity of the CA. In the third step it is outlined that the Ca^2+^-salt of polyphosphate (polyP) undergoes hydrolysis through the alkaline phosphatase (ALP), resulting in the liberation of both orthophosphate (pink filled triangles) and Ca^2+^, both components are required for the synthesis of HA [(Ca_5_(PO_4_)_3_(OH)]. Partially taken from [58] with permission.

Our experimental data show that SaOS-2 cells, exposed to bicarbonate and the MAC, form a significantly increased amount of Ca-deposits, as analyzed by a staining procedure with alizarin red S [7]. The MAC supplement (ascorbic acid, β-glycerophosphate and dexamethasone) stimulates cellular differentiation processes. Importantly, it had been measured that this process is paralleled by an enhanced expression of the CA-II gene, suggesting its participation in the Ca-deposit formation. Furthermore, the CA-II inhibitor acetazolamide significantly inhibited the Ca-mineral deposition process. These data favor the assumption that a CA-II-driven enzymatic process is involved in the formation of calcium carbonate bioseeds, required for the initial Ca phosphate deposit synthesis onto SaOS-2 cells. The CA-II is ubiquitously present in the cytoplasm of almost all metazoan cells and, focusing on mammalian bone cells, is probably involved in bone resorption [52]. There, CA-II causes proton production, resulting in a drastic acidification of the resorption lacuna/bone regions. However, recent studies implicate that this enzyme is also involved in bone formation [35]. These surprising Janus-faced catabolic/anabolic metabolic reactions, controlled by CAs might be explained on account of the reversibility of the CA-catalyzed reaction. The CA acts both as a calcium carbonate anabolic enzyme, facilitating and accelerating bicarbonate formation, a precursor molecule for calcium carbonate synthesis, and also as a catabolic enzyme that promotes calcium carbonate dissolution, as shown, e.g., in corals [53]. Experimental data revealed that during the initial phase of the controlled bone-synthesizing process poorly crystalline carbonated apatite is deposited, which contains several percents (4–6 wt %) of carbonate in the apatite crystals [54–55]. Recent studies suggest that the increased carbonate content in apatite crystals has an anabolic effect on bone formation [56]. Our EDX mapping studies [20] indicate that the crystallites initially formed onto SaOS-2 cells are not only rich in the elements calcium and phosphorous but also in carbon. We have taken this observation as a further indication that carbonate and phosphate deposits are co- or sequentially synthesized onto SaOS-2 cells, during the initial phase of mineral formation. Furthermore, the CA-II has been proven to be (under certain physiological conditions [pH regulation]) localized at the plasma membranes of human pancreatic cells [36], where the enzyme is involved both in pH regulation and in the secretion of bicarbonate through the Cl^−^/HCO^−^ exchanger [57] and/or an additional HCO_3_^−^ channel [36]. We concluded from the data gathered [20] that the calcium phosphate/HA deposition reactions in bone tissue are preceded by calcium carbonate precipitation, a process that is driven by an increased CA activity (Figure 5).

### Carbonic anhydrase: A new target for bone anabolic agents

A number of therapeutic targets have been described influencing signaling pathways, and/or transcription factors to stimulate bone growth (see [59]). Among those are the BMP/SMAD, Wnt/β-catenin, Hedgehog/Gli, IGF, and FGF pathways. Emerging evidence indicate that the CA enzyme could also be tackled as a promising target for activators to stimulate calcium carbonate/phosphate mineral deposition onto bone cells. Only very little experimental evidence has been presented that supports our supposition that CA activators exert a potential therapeutic effect on bone anabolism [60]. Until now only a few CA activators have been identified, but none of them have been tested for its potential in the treatment of bone disorders [61–63]. This view might be changed in view of the now available data indicating that calcium carbonate deposits might function as bioseeds for calcium phosphate precipitation onto bone-forming cells.

It is known that mineralization of osteoblasts, bone mineral-forming cells, is significantly induced by polyphosphate [polyP] in vitro even in the presence of orthophosphate [64]. PolyP is a linear polymer of phosphate linked by energy-rich phosphodiester bonds. Moreover, polyP turned out to be an inducer of osteoblast-specific alkaline phosphatase. This finding is interesting in view of published data indicating that intracellularly polyP might be formed in the vesicles of bone-forming cells as a Ca salt, which may act as a potential precursor of carbonated HA [65] (Figure 5).

In our recent study we could show that CA-driven CaCO_3_ deposition can be stimulated by CA activators in vitro [66]. As activator(s) we have chosen extracts from the sponge *S. domuncula* and one component, isolated from those extracts, quinolinic acid (QA). In the in vitro CA-driven calcium carbonate deposition assay we could demonstrate that the *S. domuncula* extract and QA stimulate mineral formation (Figure 3C); as controls, the assays had been performed in the absence of sponge extract or of pure QA. Furthermore the results revealed that the stimulatory effect of bicarbonate ions on mineralization onto osteoblast-like SaOS-2 cells is strongly enhanced if the cells are exposed to polyP [64]. Finally, after hydrolysis of polyP through the alkaline phosphatase, the liberated orthophosphate inhibits in a negative feedback circle the CA (Figure 5).

### Future direction: 3D printing

In the repair of critical-size bone defects, autogenous bone grafts are considered to be the gold standard [67]. This technique has, however, several limitations which cannot be solved by using allogenous bone grafts, which have additional disadvantages, such as immunogenicity and risk of infection. Synthetic bone scaffolds can provide several advantages if they meet the following conditions: (i) similar physiochemical characteristics as the natural bone, and (ii) ability to attract the bone forming cells (the progenitor cells or the functionally active differentiated cells), two challenging tasks, limiting the routine application of synthetic materials in the treatment of bone defects. Bone repair materials like calcium phosphate, calcium carbonate, calcium sulfate and coralline carbonate grafts are characterized by good mechanical properties. They can be used as osteoconductive implant materials. They also may show osteointegrative properties. However, these implants lack any osteoinductivity and must be functionalized to become biologically active (for a review, see [68]).

The prerequisite for any scaffold applicable for bone tissue engineering is that it is accepted by the cells as a suitable 3D platform for their growth, differentiation and mineralization (HA deposition). These requirements can be met by scaffolds made of natural fibers, which correspond in their structure and composition to the extracellular matrix. A suitable scaffold must possess the inorganic/organic 3D structure of bone and an appropriate porosity [69] that allows the ingrowth of cells and an efficient transport of cytokines, growth factors, and nutrients. To avoid necrosis within larger implants, a suitable scaffold must also allow an efficient vascularization and tissue supply with oxygen. Much progress has been achieved in rapid prototyping/3D printing techiques in the last years. 3D printing is a computer-controlled layer-by-layer technology. Thereby a binder (binding solution) is printed into each layer of powder, a step-wise process that finally results, after blowing-away the unbound powder, in a 3D printed copy of the sliced virtual model [70–71]. 3D printing has turned out to be of promising technique for the fabrication of implants used as bone substitution materials [72]. The advantage of this method is that the implants can be customized to the 3D geometry of the bone defect of an individual patient, based on medical imaging data. Such implants allow an optimal integration and can be provided with the required functional properties using suitable materials such as bioactive glasses and Ca phosphate.

**Figure 6:**
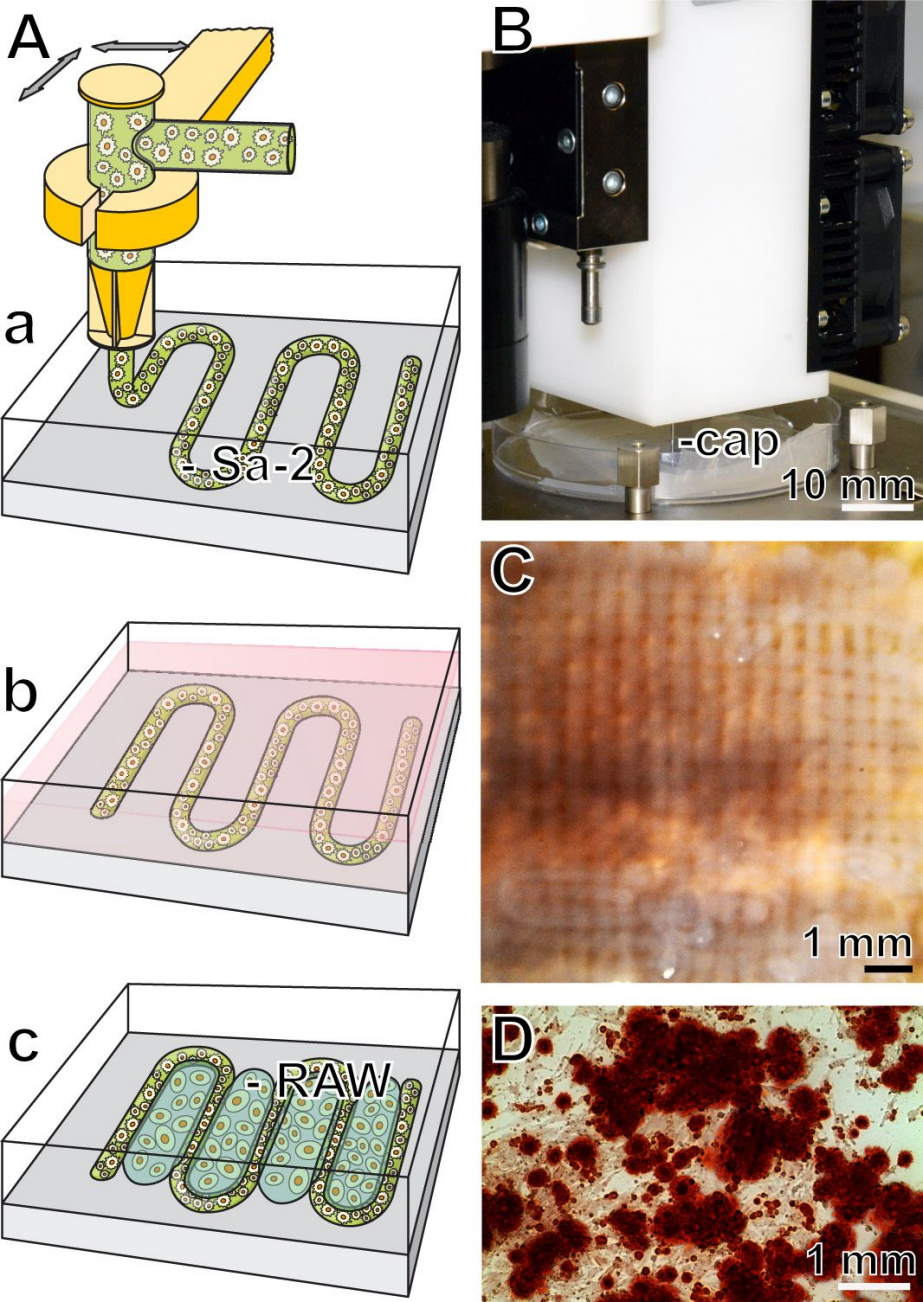
Computer-aided rapid prototyping bioprinting. (A-a) A sketch outlining the computer-guided extrusion of Na alginate hydrogel (supplemented with biosilica or bicarbonate) through a capillary in a meander-like pattern. This matrix contained SaOS-2 cells (Sa-2). (A-b and A-c) The blocks formed were incubated in medium into which RAW 264.7 cells (RAW) had been suspended. (B) Part of the bioplotter showing the capillary (cap) through which the alginate/cell matrix is extruded. (C) Completed 4 mm high blocks into which the cells have been embedded into the alginate. (D) The cells retain the capacity to form crystallites, which can be visualized after staining with Alizarin Red S.

Based on published data, indicating that alginate/chitin, also together with silica [73–74], provides a suitable matrix for the encapsulation of mammalian cells we have recently also embedded SaOS-2 cells into Na alginate that has been supplemented with silica [75–77]. Silica displays morphogenetic activity towards SaOS-2 cells (see above); this biological activity of silica is retained by SaOS-2 cells that have been encapsulated into Na alginate. Based on the finding that Na alginate is a suitable matrix for embedding bone cells [78] we have successfully started to print 3D structures in order to apply this technology for bioprinting and construction of bioartificial tissues or organs. In a first step we have encapsulated separately bone-forming (SaOS-2) and bone-degrading (RAW 264.7) cells to develop a biomimetic synthetic scaffold suitable for tissue engineering [75]. In the alginate matrix applied the SaOS-2 cells retain their capacity to synthesize HA crystallites. Furthermore, the mechanical properties, including surface roughness and hardness, of the hydrogel were determined. If silica is included in the hydrogel matrix, the encapsulated SaOS-2 cells were found to increasingly express the gene encoding for osteoprotegerin in co-cultivation experiments with RAW 264.7 cell beads, suggesting that under the applied conditions the differentiation capacity of the RAW 264.7 cells is impaired. In continuation it was found that under these conditions (SaOS-2 cells cultured together with RAW 264.7 cells) the RAW 264.7 cells show a reduced capacity to express the gene for tartrate-resistant acid phosphatase. For rapid prototyping bioprinting we are using a computer-aided tissue engineering printer (3D-Bioplotter; Corporate EnvisionTEC GmbH, Gladbeck; Germany). With this technology we succeeded to embed SaOS-2 cells into the Na alginate, with the indicated supplements, and allowed the matrix to be passed through the capillary of the 3D printer (Figure 6). The arrays of strands were computed to 4 mm high blocks into which the cells remained viable and retained the capacity to form mineral crystallites. We are convinced that this strategy will contribute to a further improvement of the formulation of a suitable artificial scaffold for rapid prototyping 3D bioprinting of organ-like tissue units.
